# Supplementary material for: Rickettsia felis and Other Rickettsia Species in Chigger Mites Collected from Wild Rodents in North Carolina, USA
Source: Microorganisms. 2022 Jul 2;10(7):1342. doi: 10.3390/microorganisms10071342 (PMC9324336; doi:10.3390/microorganisms10071342)
Supplement: Supplementary file 1 [file microorganisms-10-01342-s001.zip › Supplementary Figure S4.pdf]

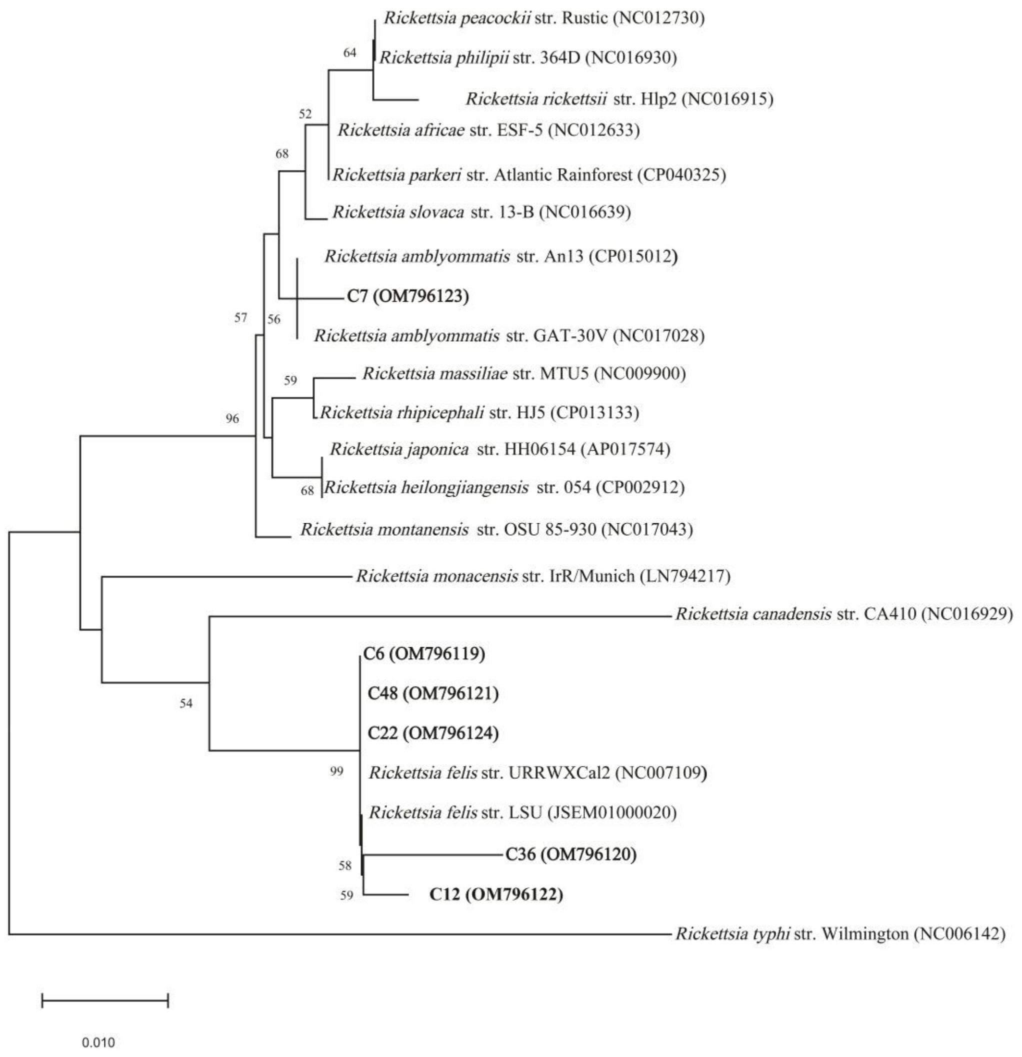

Figure S4. Phylogenetic relationships derived from partial sequences (~287 bp) of the *Rickettsia* citrate synthase (*gltA*) gene amplified from chigger DNA samples (highlighted in bold) and other related *Rickettsia* taxa. The phylogenetic tree was reconstructed using the neighbor-joining method. The GenBank accession numbers are given in the parentheses. Scale bars indicate the number of substitutions per nucleotide position.
